# Diffuse Non-Vascular Cornitis

**Published:** 1883-05

**Authors:** Chas. W. Hickman

**Affiliations:** Augusta, Ga., Lecturer on Diseases of the Eye in the Medical Department of the University of Georgia


### DIFFUSE NON VASCULAR CORNITIS.

By Chas. W. Hickman, M. D., Augusta, Ga.,

Lecturer on Diseases of the Eye in the Medical Department of the University of Georgia.

Inflammation of the cornea may be produced by a number of causes, and may occur at any age of life. In the majority of cases we meet with it in children and chiefly confined to those of a delicate constitution, or who have inherited a scrofulous or syphilitic taint. The various forms which the inflammation assumes according to the cause, is a subject of no little interest. When we consider that this beautiful yet delicate structure contains no blood vessels of its own, but is entirely dependent upon the surrounding parts for its nutrition, we can easily imagine the number of ills to which it may be exposed from a defective or impoverished blood supply. One of the first things we notice in examining a healthy cornea is its perfect transparency and high degree of polish, returning an erect but diminished image of any object placed in front of it. The minute the polished appearance becomes indistinct or lost, we may be sure that an inflammation of some kind is about to set in. This is first noticed as a hazy, steamy appearance, such as we see when we blow our breath upon a pane of glass. This steaminess may show itself at any portion of the cornea, and gradually spread over the entire surface. Most frequently it commences towards the circumference, extending inwards, the part first attacked getting well as the haziness advances. Although, in some cases, intolerance of light and pain may be complained of, yet as the rule this is not the case unless the peripheral distribution of the nerves become exposed from the inflammation involving the deeper layers to such an extent as to entirely destroy the epithelial cells. The other eye is almost sure to be effected, it may be, at the same time, but most frequently the one precedes the other, the first even getting well before the other becomes involved.

Very simple measures suffice for the treatment of diffuse kerotitis, the natural tendency of the affection being to terminate in recovery; although several weeks or even months may be required for it to run its course, as a description of one or two cases may afford a good illustration.

The first case was that of a girl 18 years of age, who, though presenting no evidence of a specific taint, yet had always been more or less delicate, scrofulous swellings making their appearance from time to time upon her neck and face. A steamy appearance of the right cornea was first seen, at the outer or lower third, and which gradually extended over the entire surface, shutting out vision almost entirely for the time. The other eye soon followed in the same way. The inflammation under very simple treatment ran its course to a successful termination in about eight weeks.

The other case was that of a child, aged four years. Here again no history could be obtained of a specific taint. nor was there any evidence present of strumous diathesis. The only thing noticed was a pale and anæmic appearance which was due, according the parents' statement, to a prolonged attack of malarial fever. The right cornea was almost entirely clouded over, a pain and intolerance of light were prominent features. Tonics were frequently given, such as the ferrated tincture of bark, with the 120th of a gr. strychnia added to each dose. Sulph. of quinine in sufficient quantities to combat the malarial symptoms, and a solution of sulph. atropia, grs 2 to the ounce, dropped into the eye three times a day, and this combined with a light compress bandage. In the course of a few weeks the cornea regained its normal transparancy and the case was dismissed as cured.

As the opaque corne amaterially interferes with sight, the first thing we have to do is to meet up the courage of our patient, and quiet his fears as to ultimate blindness, Bearing in mind that the tendency of the inflammation is towards recovery, and that we mostly meet with it in those of a delicate, strumous, or syphilitic constitution, our next indication is to build up the stamina and combat any specific taint as our judgment dictates. A solution of sulph. atropia grs 1 to 2 to the ounce of water may be dropped into the eye, as by this means we accomplish three important ends, viz: Diminish the quantity of aquous, keep the iris dilated and at rest and relieve pain. A light compress bandage should be worn during the day which keeps out the excess of light. yet at the same time does not prevent the patient from having access to an abundance of much needed fresh air.

